# Downregulation of B7-H4 suppresses tumor progression of hepatocellular carcinoma

**DOI:** 10.1038/s41598-019-51253-2

**Published:** 2019-10-16

**Authors:** Lijie Dong, Lulu Xie, Minjing Li, Hanhan Dai, Xia Wang, Peiyuan Wang, Qiang Zhang, Wei Liu, Xuemei Hu, Mingdong Zhao

**Affiliations:** 1 Department of Imaging, Binzhou Medical University, Binzhou, Shandong 264003 P.R. China; 2 Department of Immunology, Binzhou Medical University, Yantai, Shandong 264003 P.R. China; 3 Medicine & Pharmacy Research Center, Binzhou Medical University, Yantai, Shandong 264003 P.R. China; 4 Department of Oral Pathology, Binzhou Medical University, Yantai, Shandong 264003 P.R. China; 5 Department of Radiology, Binzhou Medical University Hospital, Binzhou, Shandong 256603 P.R. China

**Keywords:** Cancer, Cancer, Cancer, Cancer, Molecular biology

## Abstract

B7-H4, as a member of the B7 superfamily, was overexpressed in various types of cancers. However, the effects of B7-H4 on the aggressiveness of HCC and the underlying mechanisms have not yet been fully explored. For this purpose, B7-H4 expression was detected by Flow cytometry and Western blotting, it was highly expressed in several HCC cell lines but not in normal LO2 cell line. Knockdown B7-H4 expression induced HCC cells apoptosis by flow cytometry and colony formation assays and increased several apoptosis-related proteins, including survivin, cleaved caspase-3, cleaved caspase-7, and Bax, while the pro-growth protein survivin was reduced. Then the proliferation and cell cycle were suppressed after treated by siB7-H4. Moreover, the level of B7-H4 was significantly correlated with cell migration. *In vivo*, intra-tumor injection of siRNA targeting B7-H4 can significantly inhibited the growth of HepG2 cells in nude mice. Finally, regions of interest were manually traced on T1WI, T2WI, DWI and ADC of MR images. ADC values were increased in HCC xenografts after B7-H4 siRNA treatment. These data indicated that downregulation of B7-H4 suppressed the proliferation and migration and promoted apoptosis *in vitro* and *in vivo*. Blocking the B7-H4 channel might be a potential therapeutic strategy for HCC.

## Introduction

Hepatocellular carcinoma (HCC) is one of the most common malignancies, particularly in Asia and tropical Africa^1,2^, and is the third leading cause of death due to cancer worldwide^3^. More than 600,000 new HCC cases are annually diagnosed worldwide, and approximately 55% of these occur in China^3^. Although there are many treatment methods for HCC including surgical resection, local ablation therapy, chemotherapeutics, and trans-arterial chemoembolization,the rate of recurrence is high and its prognosis is still unsatisfactory^4,5^. Identify and develop novel approaches for the diagnosis and therapy of HCC, many researchers focus on new molecular targets and mechanisms associated with HCC.

B7-H4 is a member of the B7 protein family and negatively regulates adaptive and innate immune responses^6–8^. It is not or only slightly expressed in normal tissues^9^. However, a few studies have reported that B7-H4 is highly expressed in some human cancers such as colorectal, pancreatic carcinomas and intrahepatic cholangiocarcinoma^10–12^. The knockdown of B7-H4 is known to inhibit proliferation, invasion, and migration in a pancreatic carcinoma cell line and to increase the levels of apoptosis^11^. Therefore, B7-H4 may play a role in the development and progression of some tumors. To date, two studies have reported that soluble B7-H4 (sB7-H4) expression is upregulated in the serum of patients with HCC and that high levels of sB7-H4 are associated with an advanced clinical tumor stage^13,14^. Furthermore, an immunohistochemical approach has been used to identify B7-H4 expression in cancerous liver tissues^15,16^. However, the effects of B7-H4 on HCC development and progression still remain unclear. Therefore, we attempted to explore the relationship between B7-H4 and HCC development using short interfering RNA (siRNA)-mediated gene silencing. Furthermore we also investigated the effects of B7-H4 down-regulation on the growth of HepG2 cells in nude mice. Our data provide further insight into the influence of B7-H4 on HCC development by modulating apoptosis, proliferation, and migration.

## Results

### B7-H4 high expression in HCC cell lines

**Figure 1:**
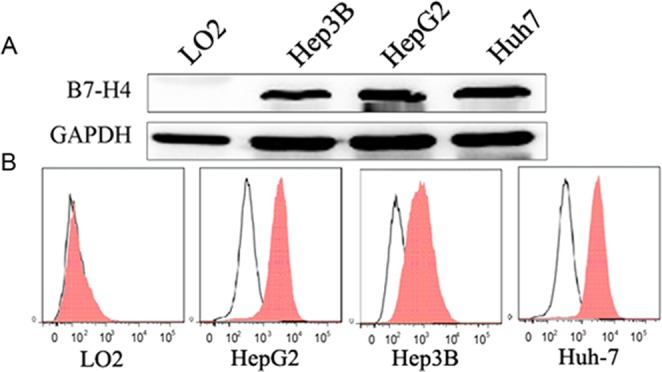
Expression of B7-H4 in a normal human liver cell line and three human HCC cell lines. (**A**) Levels of B7-H4 were determined by Western blot analysis from LO2, Hep3B, HepG2, and Huh-7 cells. (**B**) Fluorescence intensity of B7-H4 in LO2, Hep3B, HepG2, and Huh-7 cells. B7-H4 was undetectable in LO2 cells but was expressed at high levels in Hep3B, HepG2, and Huh-7 cells.

B7-H4 expression was undetectable in normal hepatocyte LO2 cells but was highly expressed in Hep3B, HepG2 and Huh-7 cells by Western blot analysis (Fig. 1A) and flow cytometry (Fig. 1B).

### Efficient siRNA-mediated B7-H4 gene silencing in HCC cell lines

**Figure 2:**
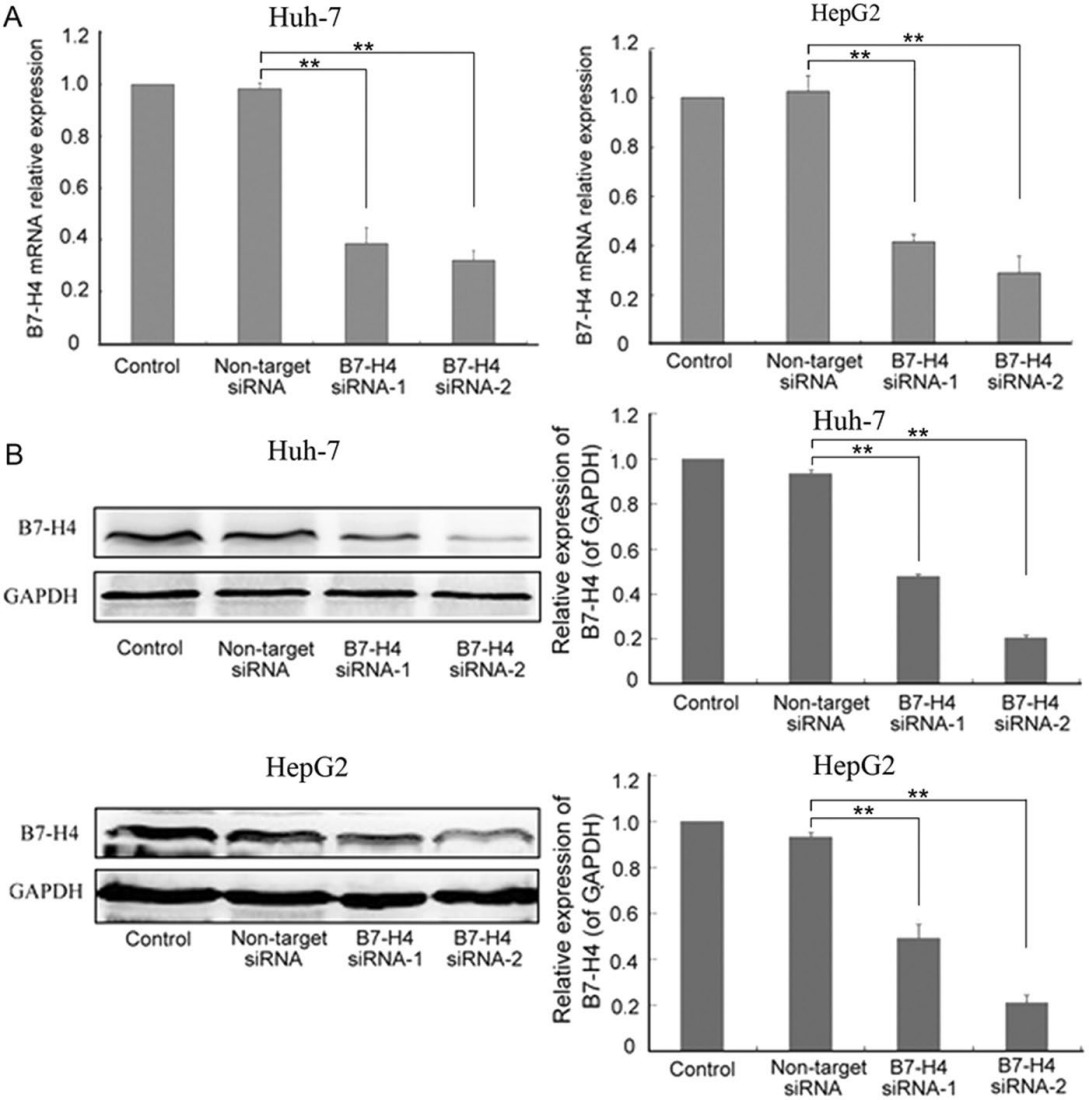
Evaluation of the effect of siRNA silencing on B7-H4 by real-time PCR and Western bloting. Both Huh-7 and HepG2 cells were transfected with either B7-H4 siRNA or non-target siRNA for 6 h. Non-transfected Huh-7 and HepG2 cells were used as controls. (**A**) After 24 h transfection, total RNA was extracted from Huh-7 and HepG2 cells in the control, non-target siRNA, B7-H4 siRNA-1, and B7-H4 siRNA-2 groups and was subjected to real-time RT-PCR analysis to determine the efficiency of siRNA transfection on the expression of B7-H4. (**B**) The four groups of cells (control, non-target siRNA, B7-H4 siRNA-1, and B7-H4 siRNA-2) were harvested and analyzed by Western blotting 72 h post-transfection to further assess the efficiency of siRNA transfection on B7-H4. ^**^p < 0.01 vs. non-target siRNA.

Real-time PCR and Western blot analysis were employed to evaluate interference efficiency. Data showed that B7-H4 mRNA expression in Huh-7 and HepG2 cells was reduced in the B7-H4 siRNA group compared with that in the non-target siRNA groups (p < 0.01, Fig. 2A). There was no significant difference in the expression of B7-H4 mRNA between the control and non-target siRNA groups (p > 0.05). The same trends were observed by Western blot analysis. The expression of B7-H4 in Huh-7 and HepG2 cells declined in the B7-H4 siRNA group compared with that in the control and non-target siRNA groups (Fig. 2B). In addition, B7-H4 expression was reduced by a more significant level in the B7-H4 siRNA-2 group than in the B7-H4 siRNA-1 group. Consequently, B7-H4 siRNA-2 was used to perform all subsequent investigations.

### B7-H4 knockdown increased the apoptotic of HCC cells

**Figure 3:**
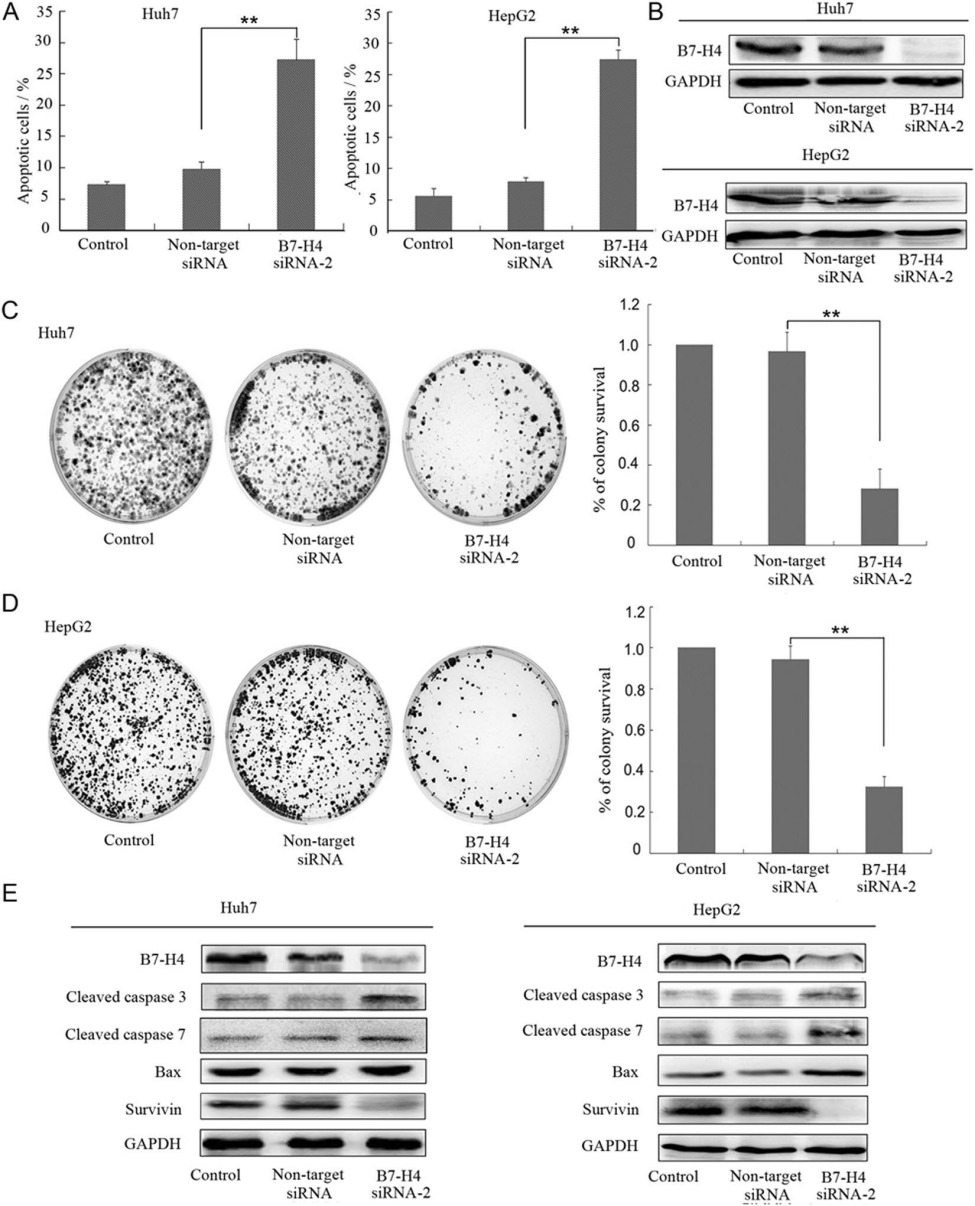
Effect of B7-H4 silencing on apoptosis in HCC cells. (**A**) Huh-7 and HepG2 cells were stained with annexin V-FITC and PI and were then analyzed by flow cytometry. (**B**) The three groups of cells (Control, Non-target siRNA and B7-H4 siRNA-2) were harvested and analyzed by Western blot 72 h post-transfection to assess the efficiency of siRNA transfection on B7-H4. (**C,D**) Effect of B7-H4 silencing on the number of colonies formed by Huh-7 and HepG2 cells. (**E**) Expression of survivin, Bax, cleaved caspase-3, and cleaved caspase-7 were analyzed in Huh-7 and HepG2 cells by Western blot analysis 72 h after siRNA-2 transfection. GAPDH was used for standardization. **p < 0.01 vs. non-target siRNA.

The apoptosis of Huh-7 and HepG2 cells was detected after 72 h in non-transfected cells and in cells transfected with siRNA, including B7-H4 siRNA-2, and non-target siRNA. The level of apoptosis in Huh-7 and HepG2 cells was higher in the B7-H4 siRNA-2 group than in the non-target groups, as measured using annexin V/PI (p < 0.01, Fig. 3A), There were no significant differences between the control and non-target siRNA group (p > 0.05). The down-regulation of B7-H4 by siRNA to B7-H4 was confirmed with Western blot (Fig. 3B). In addition, colony formation assays revealed that Huh-7 and HepG2 cells formed fewer colonies in the B7-H4 siRNA-2 group than in non-target groups (p < 0.01, Fig. 3C,D). There were no significant differences between the control and non-target siRNA group (p > 0.05). To further investigate the mechanisms of HCC apoptosis caused by B7-H4 siRNA-2 transfection, apoptosis-related proteins were analyzed by Western blot. The results showed that the levels of the pro-apoptotic proteins Bax, cleaved caspase-3 and cleaved caspase-7 were increased, but the levels of the anti-apoptotic protein survivin in the B7-H4 siRNA-2 group were decreased compared with those in the control and non-target groups (Fig. 3E).

### B7-H4 knockdown suppressed the proliferation of HCC cells

**Figure 4:**
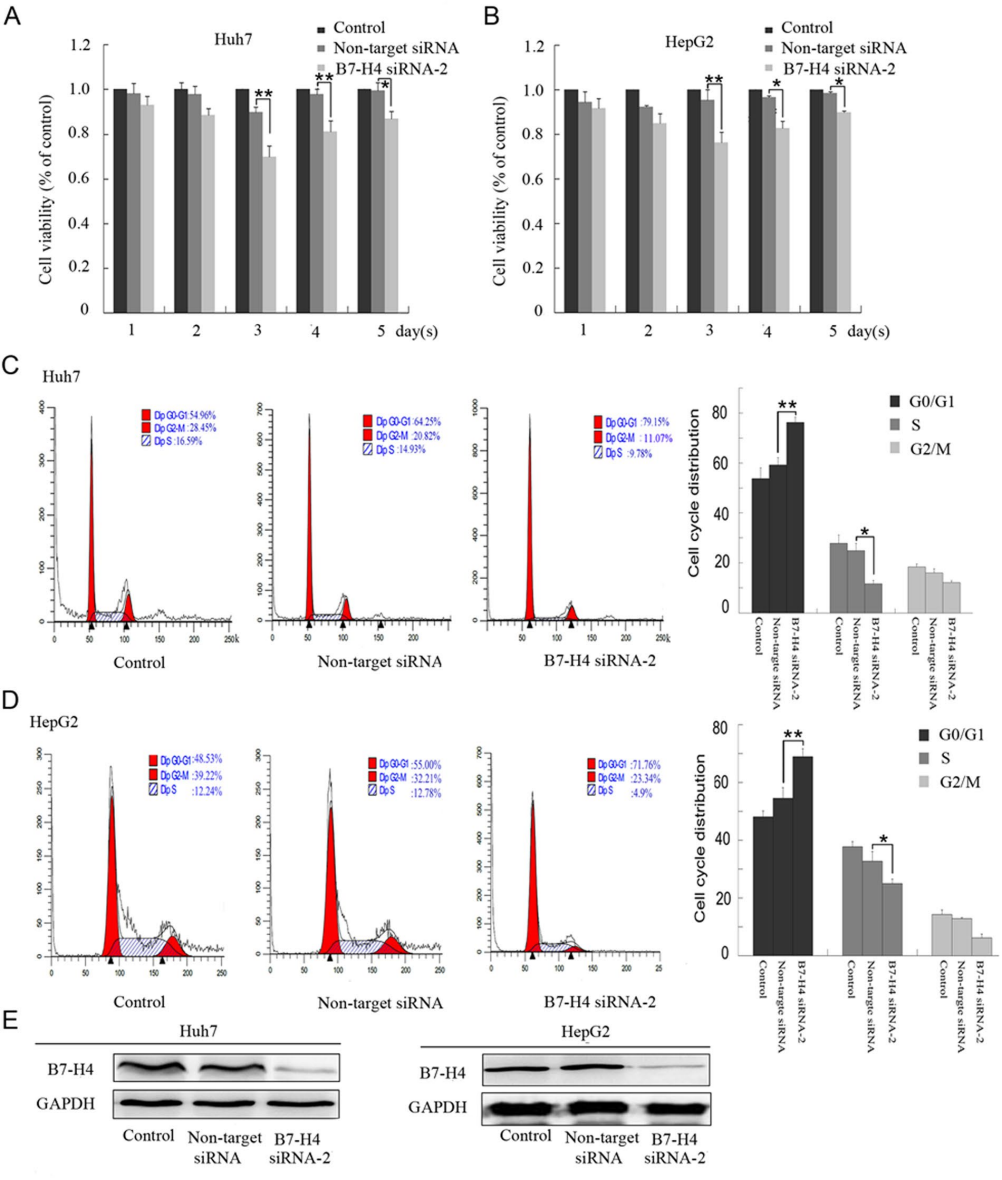
Effect of B7-H4 silencing on the proliferation of HCC cells. (**A,B**) The viability of Huh-7 and HepG2 cells was identified by the CCK-8 kit at 1, 2, 3, 4, and 5 d time-points following siRNA-2 transfection. (**C,D**) Cell cycle distribution of Huh-7 and HepG2 cells was identified by flow cytometry 72 h after siRNA-2 transfection. **p < 0.01 and *p < 0.05 vs. non-target siRNA. (**E**) The three groups of cells (Control, Non-target siRNA and B7-H4 siRNA-2) were harvested and analyzed by Western blotting 72 h post-transfection to assess the efficiency of siRNA transfection on B7-H4.

To determine whether the silencing of B7-H4 by siRNA affected the proliferation of human HCC cells, the proliferation of Huh-7 and HepG2 cells were evaluated using a cell proliferation assay. The growth of Huh-7 cells was significantly inhibited in the B7-H4 siRNA group at 3 d (p < 0.01), 4 d (p < 0.01), and 5 d (p < 0.05) time-points in comparison with that in the non-target siRNA groups (Fig. 4A). Similarly the growth of HepG2 cells was reduced in the B7-H4 siRNA group at 3 d (p < 0.01), 4 d (p < 0.05), and 5 d (p < 0.05) time-points (Fig. 4B). The growth of both cell lines were no significant differences between the control and non-target siRNA group (p > 0.05). Furthermore, the cell cycle was determined at 72 h following B7-H4 siRNA-2 transfection by flow cytometry. Most Huh-7 and HepG2 cells were arrested in the G0/G1 phase in the B7-H4 siRNA-2 group compared to the control and non-target siRNA group (Fig. 4C,D). In parallel, cell lysates from control, non-target, or B7-H4 siRNA-transfected cells were harvested and subjected to Western blot analysis of B7-H4 expression (Fig. 4E).

### B7-H4 knockdown suppressed the migration of HCC cell lines

**Figure 5:**
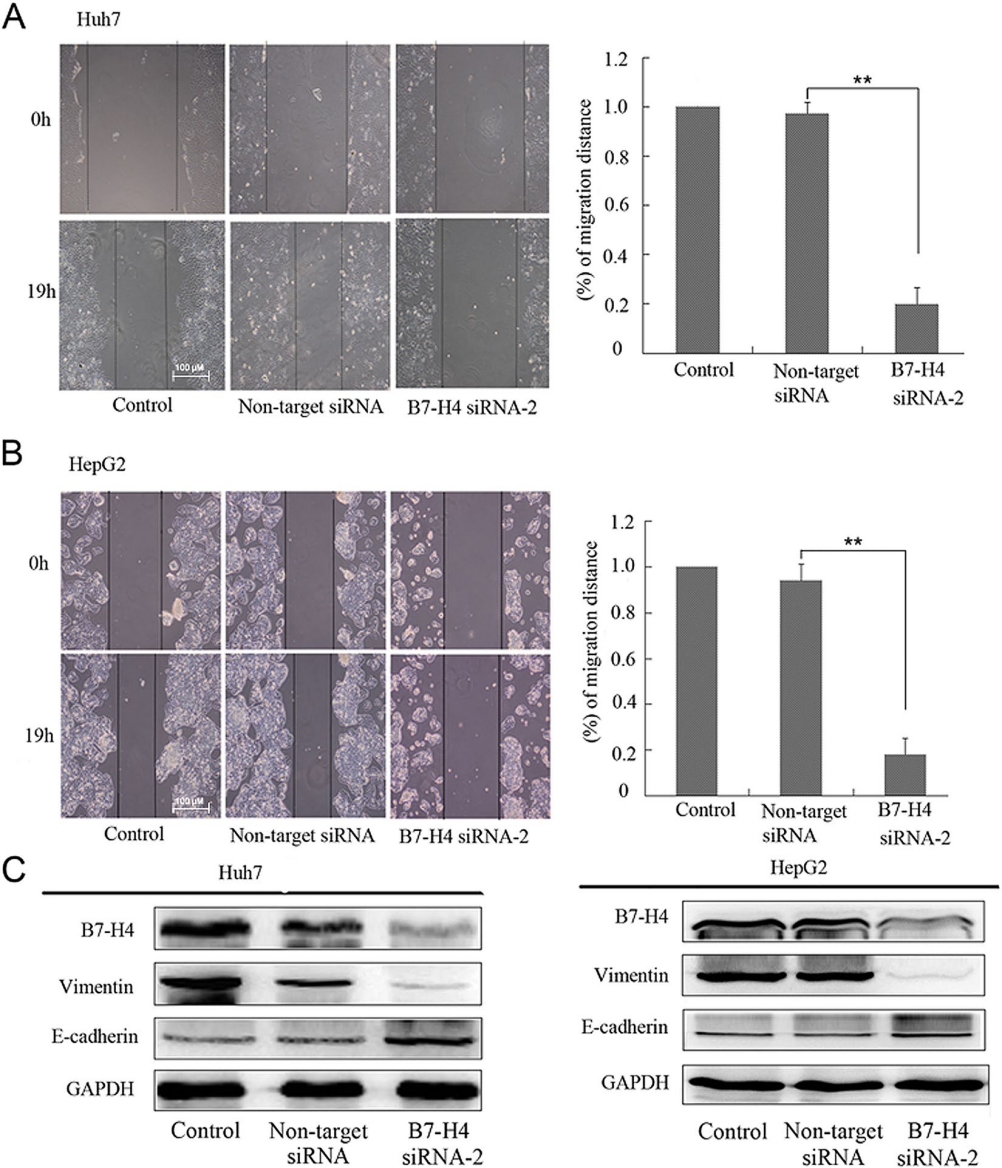
Effect of B7-H4 silencing on HCC cell migration. (**A,B**) To evaluate the migration ability of Huh-7 and HepG2 cells at 72 h after siRNA transfection, scratch assays were performed for 0 h and 19 h. (**C**) To further evaluate the relationship between B7-H4 and HCC cell migration, the expression of E-cadherin and vimentin was analyzed in Huh-7 and HepG2 cells by Western blot analysis 72 h following siRNA-2 transfection. GAPDH was used for standardization. **p < 0.01 vs. non-target siRNA.

The wound healing capacity of Huh-7 and HepG2 cells at 72 h in the B7-H4 siRNA-2 group were reduced compared with that in the non-target groups (p < 0.01, Fig. 5A,B), but there were no significant differences between the control and non-target siRNA group (p > 0.05). Western blot was used to examine the migration-related proteins E-cadherin and vimentin. The E-cadherin expression level of Huh-7 and HepG2 cells were all increased in the B7-H4 siRNA-2 group whereas vimentin were decreased comparied with non-target siRNA group, but there were no significant differences between the control and non-target siRNA group (Fig. 5C).

### B7-H4 knockdown suppressed human HCC growth ***in vivo***

**Figure 6:**
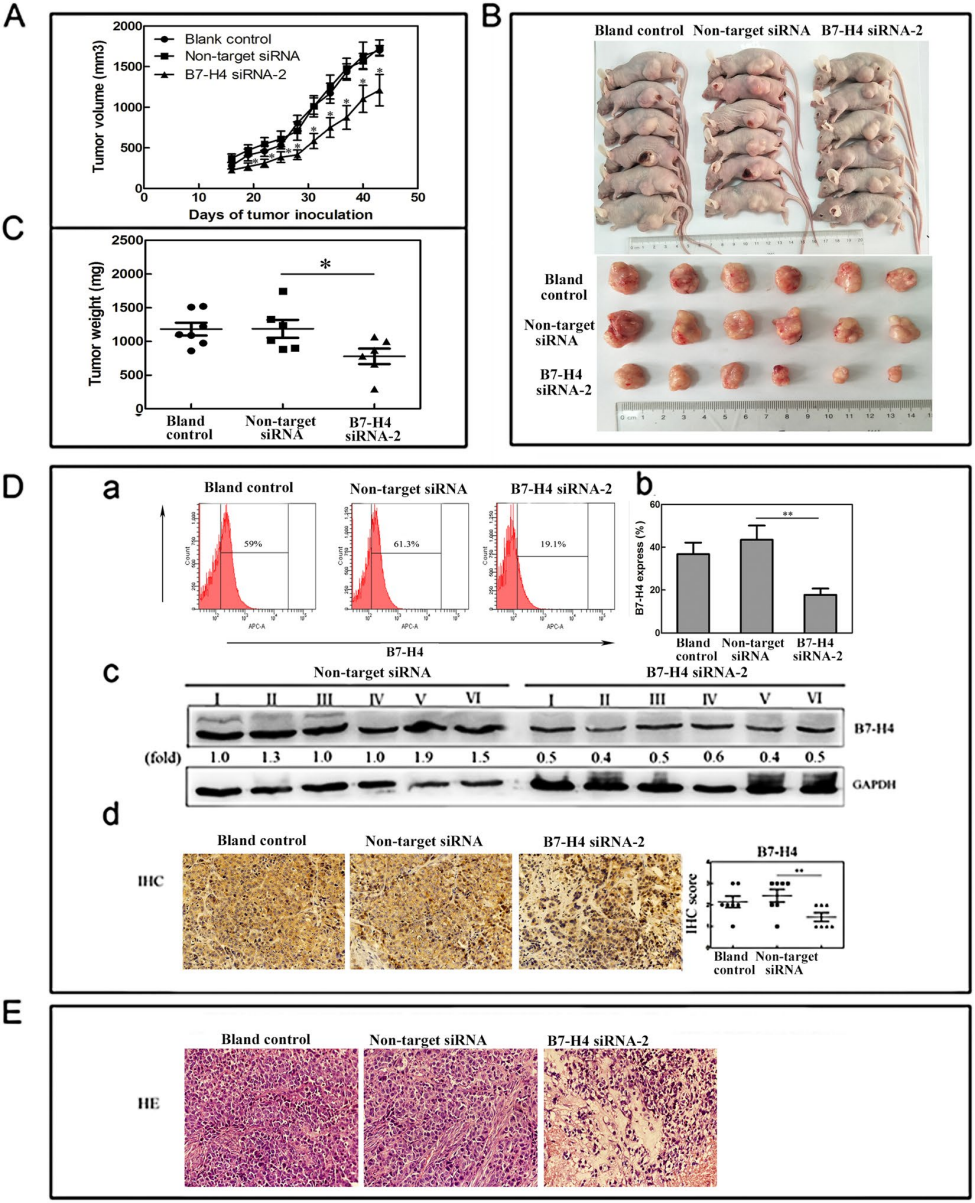
Effect of chemically modifed B7-H4 siRNA on tumor growth on HCC xenografts. (**A**) Tumors were measured weekly during the experimental period. (**B,C**) Tumors were surgically excised and weight. (**D**_a–d_) Flow cytometry western blot and IHC were used to detect B7-H4 expression in the excised mouse tumor tissues.**p < 0.01 and *p < 0.05 vs. non-target siRNA. (**E**) Original HE images of the Blank control group, Non-target siRNA and B7-H4 siRNA-2 group (magnification ×400).

Tumors volumes of three groups were measured twice a week and drew the tumor growth curves. The tumor growth curves indicated that there was a significant growth inhibition in B7-H4 siRNA2 group (P < 0.05), but no differences of blank control group and non-target siRNA group (P > 0.05) after treated with multi-point injection of siRNA for 4 weeks (Fig. 6A). The tumor volume of three groups were measured at 6 weeks, the result showed that the B7-H4 siRNA-2 group were 1211 ± 193.1 mm^3^, 1730 ± 98.57 mm^3^ in the non-target siRNA group and 1700 ± 58.86 mm^3^ in the blank control group, while the corresponding weight were 777.7 ± 114.0 mg, 1186 ± 133.5 mg and 1181 ± 95.73 mg respectively. The tumor volume and weight of the B7-H4 siRNA-2 group were obviously reduced compared with that in non-target siRNA group (p < 0.05, p < 0.05), but there was no difference between non-target siRNA group and blank control group (Fig. 6B,C). B7-H4 expression was detected in xenografts of each group by flow cytometry, that the level of B7-H4 expression was lower in the B7-H4 siRNA-2 group than that in the non-target groups (p < 0.01, Fig. 6D_a_), but no differences between non-target siRNA group and blank control group (P > 0.05). The down-regulation of B7-H4 by siRNA to B7-H4 was further confirmed with western blot and IHC (Fig. 6D_b,c_). The results of HE stain demonstrated a decrease in cell density and an increase in degree of necrosis in tumors of B7-H4 siRNA group (Fig. 6E).

### MRI features of HCC xenografts

**Figure 7:**
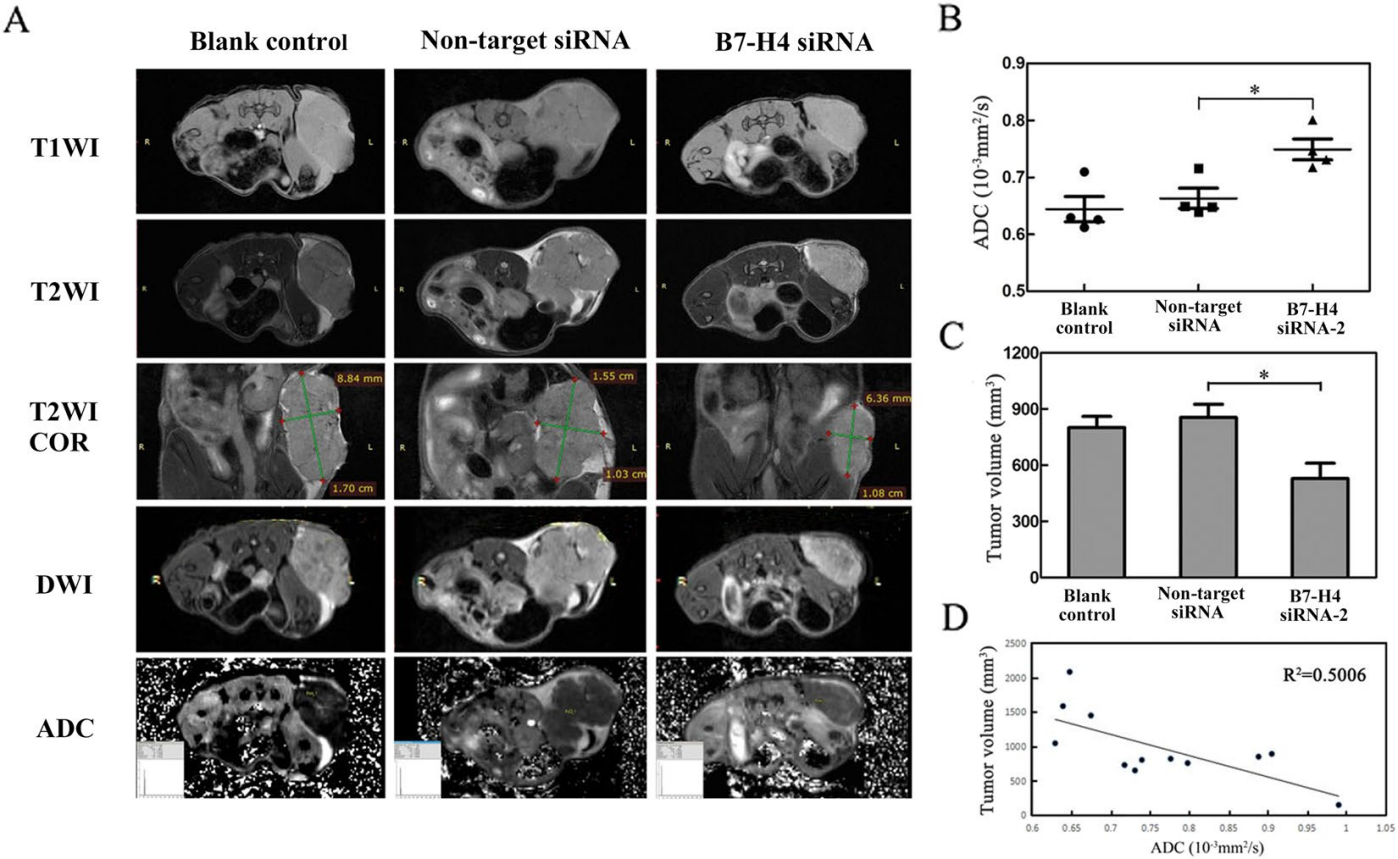
MRI of HCC xenografts. (**A**) The rows represent the three groups maps, Blank control group (left), Non-target siRNA group (middle) and B7-H4 siRNA-2 group (right). The columns represent the sequence, T1WI, T2WI, coronal T2WI plus fat repression, DWI and ADC Maps. Matching features in the vivo images, identified by visual inspection. The Lenght and Widht of tumors were measured by the straight line on coronal T2WI. The displayed image FOV is 40 × 40 mm. (**B**) DWI parameter for the Blank control group, Non-target siRNA and B7-H4 siRNA-2 groups. ADC = mean apparent diffusion coefficient. *p < 0.05 vs. non-target siRNA. (**C**) The tumor volume of Blank control group, Non-target siRNA and B7-H4 siRNA-2 groups acquired on coronal T2WI *in vivo*. (**D**) Relationship between the tumor volume and the ADC values.

T1WI, T2WI, DWI could provide additional information about HCC xenograft tumors, such as position, size, shape, abnormal regions and intensity of tumors *in vivo*. All tumors and adjacent organ of three groups were detected clearly on T1WI and T2WI images, observed isointensity on T1WI images, hyperintensity and more hyperintensity in necrotic areas on T2WI; In addition, the B7-H4 siRNA-2 group exhibited a major decrease of tumor volume on Coronal T2WI plus fat repression compared with non-target siRNA group and blank control group. DWI images showed hyper-intensity and more hyperintensity in solid lesions of tumor tissues. For ADC maps, the solid of tumor lesions of three groups were clearly exhibited low signal intensity (Fig. 7A). Furthermore, DWI related parameters, ADC values were measured on ADC maps, Data showed that the ADC values were decreased in the B7-H4 siRNA group compared with that in the non-target siRNA groups (p < 0.05, Fig. 7B), There was no significant difference between the control and non-target siRNA groups (P > 0.05). The tumor volume of the B7-H4 siRNA-2 group acquired on coronal T2WI *in vivo* were obviously reduced compared with that in non-target siRNA group (p < 0.05, Fig. 7C), but there was no difference between non-target siRNA group and blank control group (P > 0.05). ADC values of three groups were all negatively correlated with their corresponding tumor volume (Fig. 7D).

## Discussion

HCC is a common malignant tumor with high morbidity and mortality, particularly in China^17^. Compared with other cancers, risk factors for HCC are relatively well known; however, the mechanisms of hepatocarcinogenesis are not well understood at present^18^. Some studies have suggested that B7-H4 is involved in the developmental mechanisms of some types of cancer including pancreatic and breast cancers^11,19^. Previous studies have reported that sB7-H4 is expressed at high levels in the serum and is associated with an advanced clinical tumor stage in patients with HCC^13,14^. In addition, the expression of B7-H4 has been detected in cancerous liver tissues^15^. However, to date, this expression remains to be quantified. The mechanism underlying B7-H4 involvement in HCC development and progression also remains to be elucidated. In the present study, we discovered that B7-H4 was highly expressed in Hep3B, HepG2, and Huh-7 cells but was undetectable in normal human hepatocyte cells (LO2). These results strongly suggest that high levels of B7-H4 expression are involved in the molecular physiology of HCC and that this may represent an important mechanism in the development and progression of HCC.

A previous report has shown that B7-H4 plays a role in cell apoptosis in pancreatic and ovarian cancers^11,20^. In the present study, flow cytometry was used to determine whether B7-H4 influenced the apoptosis of human HCC cells. The results showed that the silencing of B7-H4 led to the induction of cell apoptosis. To further verify the anti-apoptotic effect of B7-H4, we quantified colony formation and found lesser number of colonies in the B7-H4 siRNA group than in the control and non-target groups. These results demonstrated that B7-H4 exerts an anti-apoptotic effect on HCC cells, and consequently promotes HCC development. Previous studies have shown that survivin is expressed at high levels in HCC cells, which could inhibit cancer cell apoptosis^21,22^. However, whether B7-H4 exerts an apoptotic effect on HCC cells by disrupting the expression of survivin remains to be elucidated. In the present study, we found that survivin was significantly downregulated in HCC cells following B7-H4 siRNA transfection. This result suggests that B7-H4 interfered with the expression of survivin and thereby inhibited HCC cell apoptosis. In addition, survivin exerts its strong anti-apoptotic effects by altering the caspase-3–caspase-7 and Bax pathways^23,24^; thus, it plays an important anti-apoptotic role. Our current results also showed that the levels of cleaved caspase-3, cleaved caspase-7, and Bax were significantly increased in HCC cells transfected with B7-H4 siRNA. Collectively, these results suggested that B7-H4 alters the expression of survivin, which can subsequently inhibit the caspase-3–caspase-7 and Bax pathways; eventually, apoptosis in HCC cells is blocked.

Some studies have reported that B7-H4 plays a role in cell proliferation in pancreatic and ovarian cancers^11,16^. Shiraki *et al*. further reported that survivin promotes the aberrant proliferation and cell cycle progression of HCC cells^21,25^. In the present study, we found that the downregulation of B7-H4 could significantly suppress the growth of HCC cells by arresting the cell cycle at the G0/G1 phase. Therefore, these data suggested that B7-H4 plays an important role in promoting HCC proliferation by regulating the function of survivin.

An earlier study demonstrated that B7-H4 promotes the invasion and migration of colorectal cancer cells^10^. However, whether B7-H4 is linked to HCC cell migration is an issue that requires further exploration. In the present study, the effect of silencing B7-H4 on HCC cell migration was determined by a cell wound scratch assay, the results of which showing that the silencing of B7-H4 could inhibit HCC cell migration. These results suggested that B7-H4 plays an important role in HCC cell migration, affecting the metastasis of HCC and resulting in HCC progression. Evidence in support of the fact that B7-H4 influences migration-related proteins including E-cadherin and vimentin and then enhances the migratory and invasive potential of pancreatic cancer has been increasing^11^. In the current study, we also evaluated the expression of E-cadherin and vimentin to investigate the potential role of B7-H4 in HCC cell migration. Our results revealed that the expression of E-cadherin increased when B7-H4 expression was blocked, whereas vimentin levels decreased. These data demonstrated that B7-H4 could induce the progression of HCC by reducing the adhesion of HCC cells and by subsequently increasing their potential migration.

Our above results demonstrate that B7-H4 plays an important role in HCC cells proliferation, invasion, and cell apoptosis. Furthermore, a strong anti-tumor effect of chemically modified B7-H4 siRNA *in vivo* was observed, as tumor growth in nude mice with xenograft was significantly suppressed when the expression of B7-H4 was down-regulated by intra-tumor injection of B7-H4 siRNA. Our results were consistent with the findings of Zhu *et al*., injected with B7-H4 siRNA inhibits tumor growth in nude mice in pancreatic cancer^26^. In addition, silencing of B7-H4 which stably transfected SMMC7721 cells with B7-H4 shRNA could inhibits tumorigenicity in HCC^27^. These results indicate that the aberrant expression of B7-H4 have many important roles in the development and progression of hepatocellular carcinoma. To further investigate the response of the HCC xenografts after B7-H4 siRNA treatment, we collected tumor xenografts for pathologic and histological analysis by means of HE. Our study showed that the tumor cell density was obviously decreased, the necrosis area was enlarged, the extracellular space was widened and cell swelling was aggravated after B7-H4 siRNA therapy by the analysis of HE staine. These results indicate that the aberrant expression of B7-H4 have many important roles in the development and progression of hepatocellular carcinoma *in vivo*.

In clinic, MRI was widely used to diagnose the several tumors included HCC^28^. MRI can dynamic track and judge the tumor development in living animal. In present study the advanced 7.0T MRI was firstly used to explore the influence of B7-H4 siRNA on progression of HCC. The T1WI, T2WI and DWI of this study were obtained on a 7.0-T MRI scanner in HCC nude mice model. The xenograft tumors of three groups were all clearly exhibited on T1WI. Moreover, we acquired the volume of tumors of the three groups on coronal T2WI *in vivo*, the tumor volume of B7-H4 siRNA-2 group were obviously reduced compared with that in non-target siRNA group and blank group, the result was consent with vernier caliper *in vitro*. In addition, DWI is a powerful sequence of quantitative assessment of the water diffusion behavior to evaluate therapeutic efficiency of targeted therapy in HCC^29^. ADC of DWI related parameter, which is a quantitative biomarker of water diffusion in the microscopic environment, can be used to detect benign and malignant lesions, assess tumor invasive potential and motility, and even predict early treatment responses^29–31^. Some studies have reported that ADC values significantly increased after days of drug treatment in triple-negative breast-cancer (TNBC) and hepatocellular carcinoma xenograft nude mice model^32,33^. However, the effect B7-H4 siRNA on HCC progression was analyzed with ADC value of MRI was still not reported. In our study, the ADC values were increased in the HCC tumor xenografts of B7-H4 siRNA mice compared with that non-target siRNA and blank control mice. Previous studies also showed that ADC values were positive correlated with the extent of tumor cell apoptosis and negative with tumor cell proliferation in nude xenograft model^32^. So the increase of ADC values in B7-H4 siRNA group indicated that tumor cell apoptosis was up-regulated, while tumor cell proliferation was down-regulated. Also, ADC values of three groups were all negatively correlated with their corresponding tumor volume in this study. These results declared that silence of B7-H4 can suppresses HCC tumor progression, obviously B7-H4 plays an important role in development of HCC.

## Conclusion

In summary, siRNA targeting B7-H4 can inhibit the growth of HCC *in vitro* and *in vivo*. The results of the study illuminate that the increase expression of B7-H4 on HCC enhance their proliferation and migration but inhibit their apoptosis. So, blocking the B7-H4 expression might be used as a potential target molecule for HCC therapy.

## Materials and Methods

### Cell culture

Human HCC lines including HepG2, Hep3B, and HuH-7 and a normal human liver cell line (LO2) were obtained from the Cell Research Institute of the Chinese Academy of Sciences (Shanghai, China); they were cultured in DMEM (Hyclone) supplemented with 10% fetal bovine serum (FBS), 100 U/mL penicillin, and 100 μg/mL streptomycin. All cells were incubated at 37 °C in a humidified atmosphere containing 5% CO_2_.

### Antibodies

Allophycocyanin (APC)-conjugated mouse antibodies specific to human B7-H4 were purchased from BD Pharmingen (USA), and rabbit monoclonal antibodies specific to B7-H4 (cat# ab108336) and Bax (cat# ab32503) were obtained from Abcam (USA). Rabbit antibodies specific to E-cadherin (cat# 3195), vimentin (cat# 5741), cleaved caspase-3 (cat# 9664), cleaved caspase-7 (cat# 8438), and survivin were provided by Cell Signaling Technology (USA). HRP-conjugated rabbit anti-D-glyceraldehyde-3-phosphate dehydrogenase (GAPDH) (cat# AB-M-M001) monoclonal antibodies were purchased from Hangzhou Goodhere Biotechnology Co. Ltd. (China), and peroxidase-conjugated AffiniPure goat anti-rabbit IgG antibodies (cat#ZB-2301) were purchased from Zhongshan Jinqiao (China).

### Cell lysis

Cells were harvested and washed twice with PBS, lysed in ice-cold radio immunoprecipitation assay buffer (Beyotime, Shanghai, China) containing a freshly prepared 0.01% protease inhibitor cocktail (Beyotime), and incubated on ice for 40 min. A cell lysis solution was centrifuged at 12,000 g for 20 min at 4 °C, and the supernatant was harvested. Protein concentrations were determined with a BCA protein assay kit (Beyotime, Shanghai, China) using bovine serum albumin as a standard.

### Western blot analysis

Cell lysates were denatured for 5–10 min at 99 °C with an SDS-polyacrylamide gel electrophoresis (SDS-PAGE) sample buffer separated on 12% SDS-PAGE gels and were transferred onto polyvinylidene fluoride membranes (Solarbio, Beijing, China). After blocking in 5% non-fat milk, membranes were incubated overnight with 1:800 primary antibodies (B7-H4, GAPDH, Bax, E-cadherin, vimentin, survivin, cleaved caspase-3, and cleaved caspase-7) at 4 °C and then with a goat anti-rabbit secondary antibody for 2 h at room temperature. For each membrane, band intensity was analyzed using enhanced chemiluminescence reagents (Thermo Fisher Scientific, Shanghai, China).

### siRNA oligonucleotides

Two sets of siRNA were prepared, as previously described^11^. Two B7-H4 siRNA oligonucleotides were synthesized by GenePharma (Shanghai, China). Negative control siRNA (non-target siRNA) was also purchased from GenePharma. Oligonucleotide sequences of siRNA are listed in Table 1.

**Table 1:** Short interfering RNA (siRNA) oligonucleotides specific to B7-H4.

siRNA	Oligonucleotide
B7-H4 siRNA-1	5′-GCU GGA GCA AUU GCA CUC AUC AUU G (dTdT)-3′ 5′-CAA UGA UGA GUG CAA UUG CUC CAG C(dTdT)-3′
B7-H4 siRNA-2	5′-GGG AGA CAC UCC AUC ACA GUC ACU A (dTdT)-3′ 5′-UAG UGA CUG UGA UGG AGU GUC UCC C(dTdT)-3′

### Transfection with siRNA oligonucleotides

Huh-7 (3 × 10^5^/well) and HepG2 (1 × 10^6^/well) cells were seeded in 6-well plates 24 h prior to transfection. When cells reached 70%–80% confluency in each well, they were transfected with 50 nM B7-H4 siRNA or a negative control using 7.5 µL Lipofectamine 2000 (Invitrogen, Shanghai, China) in accordance with the manufacturer’s protocol and were cultured in DMEM without FBS for 4–6 h. Then, the cells were cultured in DMEM with 10% FBS. All experiments were performed in triplicate. After 24 h, the transfected and non-transfected cells were collected and evaluated for mRNA levels by quantitative real-time PCR. After 72 h, the cells were collected and evaluated for protein levels by flow cytometry (BD, FACSCanto^TM^II) and Western blot analysis.

### Real-time PCR

RNA was extracted with the Trizol reagent (Takara Biotechnology Inc., Japan) from the transfected and non-transfected cells and was then reverse transcribed with a PrimeScriptTM RT-PCR Kit (Takara Biotechnology Inc.) according to the manufacturer’s instructions. Table 2 provides a list of all target genes with their corresponding primers (synthesized by Shanghai Sangon Biotech Co.) used for real-time PCR. Real-time PCR was performed using a SYBR Green real-time PCR reagent (Roche) in a total volume of 20 μL under the following conditions: 10 min at 96 °C followed by 40 cycles at 95 °C for 15 s and at 60 °C for 60 s. The β-actin gene was used for normalization. A comparative CT method (2^−∆∆CT^) was applied to quantify gene expression.

**Table 2:** Primers used in real-time PCR.

Gene	Primer sequence	Species	Amplicon size (bp)
B7-H4	Forward:5′-AGGCTTCTCTGTGTGTCTCTTC-3′	Human	227
	Reverse:5′-CTTGCTCTTGTTTGCTCACTCC-3′	Human	
β-actin	Forward:5′-TTGTTACAGGAAGTCCCTTGCC-3′	Human	101
	Reverse:5′-ATGCTATCACCTCCCCTGTGTG-3′	Human	

### Flow cytometry analysis

Each cell sample (1 × 10^6^) was incubated with the APC-conjugated B7-H4 protein for 30 min at 4 °C. The cells were washed twice and were analyzed by flow cytometry.

### Apoptosis assay

Huh-7 and HepG2 cells were divided into three groups: control (untransfected group), non-target siRNA-transfected group, and B7-H4 siRNA-2-transfected group. These cells were transfected with siRNAs for 72 h. Apoptosis assays were performed using an annexin V-FITC/PI detection kit (KeyGEN Biotech Co. Ltd, Nangjing, China). Briefly, the three groups of cells were stained with annexin V and PI according to the manufacturer’s instructions. Fluorescence signals from at least 10,000 cells were evaluated using a flow cytometer, and the apoptosis rate was immediately determined by flow cytometry.

### Colony formation assay

Following transfection, Huh-7 and HepG2 cells were counted and seeded in 6-well plates at a density of 5,000 cells per well. The plates were incubated at 37 °C and 5% CO_2_ in a humidified incubator. The culture medium was replaced every 4 days. After 12 days of culture, cells were stained with Giemsa (Solarbio, Beijing, China), and the number of colonies was counted.

### Cell proliferation assay

Cell viability was assessed with Cell Counting Kit (CCK)-8 (Tongren, Shanghai, China). Briefly, Huh-7 and HepG2 cells (4 × 10^3^/well) were seeded in each 96-well plate and were then transfected with siRNAs. They were incubated for 1–5 days. CCK-8 was added to each well, and cells were incubated for 3 h at 37 °C. Optical density (OD) at 450 nm in each well was measured using a microplate reader. All experiments were performed in triplicate.

### Cell cycle assay

Seventy-two hours after transfection, Huh-7 and HepG2 cells were harvested. Cell cycle assays were performed using cell cycle detection kit (KeyGEN, Nangjing, China) and all operations according to the manufacturer’s instructions. Cell cycle distribution was analyzed using a flow cytometer. DNA histograms were analyzed by ModFit LT 4.1.

### Cell wound scratch assay

Huh-7 and HepG2 cells were transfected with siRNA and incubated for 72 h. A 200-μL plastic tip was then used to damage cell monolayers. Then, cell migration into the wounded area was measured at 0 h and 19 h using an Olympus IX41 microscope at a 10× magnification. The wound area in each micrograph was estimated by outlining the remaining wound, and the area was measured using Image-Pro Plus 6.0. The average wound width was determined by dividing the wound area by the length of the analyzed region.

### ***In vivo*** tumor growth and treatment

Female Balb/c athymic nude mice aged 4 weeks were obtained from Vital River Laboratories (Beijing, China) and allowed one week of acclimatization to their new surroundings. Then these mice were housed in temperature-controlled rooms with a 12 h alternating light–dark cycle of Specific Pathogen Free animal laboratory. As some previous studies described HepG2 cells could form subcutaneous tumors in nude mice^34,35^. HepG2 cells (1 × 10^7^) were injected subcutaneously into the dorsal region near the hind leg of the nude mice, when the tumor tumor volumes reaching approximately 100 mm3, 18 mice with equivalently sized tumors were randomized into three groups. Animals were treated with an intratumoral multi-point injection every 3 days with 25 ul PBS (Blank control group) or with complexes of 15 μg siRNA, a set of 2′-o-Me and 5′ cholesterol-modified B7-H4 siRNA-2 (B7-H4 siRNA-2 treatment group) or negative siRNA (Non-target siRNA treatment group) together with 5 ul Lipofectamine 2000 respectively as previous studies described^36,37^. Tumors were measured twice a week and tumor volumes were calculated by using the formula: volume (A × B^2^)/2, where A is the larger and B is the smaller diameter. After 4 weeks, All mice were killed after Magnetic resonance imaging (MRI), and tumors were collected for histological analysis. Serial section of tumor tissues were stained with hematoxylin and eosin (H&E) and immunohistochemistry (IHC) as studies previously described^12^. Briefly, immunostaining analysis was independently performed by two pathologists. Five fields were randomly selected per sample, and staining intensity of tumor cells was assessed. The intensity of staining was scored as follows: 0 (negative), 1 (weakly positive), 2 (moderately positive) or 3 (strongly positive).

### Ethics statement

This study was carried out in strict accordance with the recommendations in the Guide for the Care and Use of Laboratory Animals of Binzhou Medical University. This research protocol was assessed and approved by the Committee on the Ethics of Animal Experiments of Binzhou Medical University (SYXK 2013 0020). All experimental procedures were performed under sodium pentobarbital anesthesia to minimize the suffering of laboratory animals.

### MRI examination

MR images were acquired using a high field 7.0 Tesla small animal scanner (Bruker BioSpec 70/20USR; Germany). Baseline Magnetic Resonance Imaging (MRI) included T1-weighted imaging (T1WI), T2-weighted imaging (T2WI), Diffusion-weighted imaging (DWI) and apparent diffusion coefficient (ADC). The MRI frame consisted of a nonmagnetic stereotactic wrist coil with a cylindric surface coil (5 cm internal diameter) positioned directly over the mouse pelvis. T1-weighted multiple slice multiple echo plus fat saturation images were performed the following parameters: repetition time (TR), 194.9 ms; echo time (TE), 2.6 ms; section thickness, 1 mm, 19 slices; matrix, 320 × 320. T2WI plus fat saturation images were performed the following parameters: TR, 1986.5 ms; TE, 34.4 ms; section thickness, 1 mm 15 slices; matrix, 512 × 512. DWI was obtained by respiratory-triggered single-shot echo with b-values of 650 s/mm^2^ and spectral presaturation with inversion recovery for fat saturation, the following parameters: TR, 2500 ms; TE, 33 ms; section thickness, 1 mm, 15 slices; matrix, 128 × 128. Three groups of HepG2 (six mice per group) tumor-bearing nude mice were prepared as follows: mice were anesthetized with 1–2% inhaled isoflurane anesthesia (ruiward life technology limited company, shenzhen).

### Statistical analysis

All results are presented as mean ± SD from at least three independent experiments, and data were analyzed with the SPSS 17.0. Simple comparisons between two groups were subjected to independent t-tests (two-tailed), and differences were considered statistically significant at p < 0.05. Statistically significant data are indicated by asterisks (*p < 0.05, **p < 0.01).

## Data Availability

All the data generated and analysed during the current study are included in this article, and are available from the corresponding author on reasonable request.
